# Investigation of the transport and absorption of *Angelica sinensis* polysaccharide through gastrointestinal tract both *in vitro* and *in vivo*

**DOI:** 10.1080/10717544.2017.1375576

**Published:** 2017-09-18

**Authors:** Kaiping Wang, Fang Cheng, Xianglin Pan, Tao Zhou, Xiqiu Liu, Ziming Zheng, Li Luo, Yu Zhang

**Affiliations:** a Hubei Key Laboratory of Nature Medicinal Chemistry and Resource Evaluation, Tongji Medical College, Huazhong University of Science and Technology, Wuhan, China;; b Union Hospital of Tongji Medical College, Huazhong University of Science and Technology, Wuhan, China

**Keywords:** *Angelica sinensis*, polysaccharide, oral administration, near-infrared fluorescence imaging, endocytosis

## Abstract

To investigate the absorption and delivery of ASP in gastrointestinal (GI) tract, cASP was successfully synthesized by chemically modifying with succinic anhydride and then conjugating with a near infrared fluorescent dye Cy5.5. Then, the capacity of oral absorption of cASP was evaluated. The results demonstrated that cASP had low toxicity and no disruption on the integrity of cell membrane. The endocytosis of cASP into the epithelial cells was time- and energy-dependent, which was mediated by macropinocytosis pathway and clathrin- and caveolae (or lipid raft)-related routes. Otherwise, the actin filaments played a relatively weak role at the same time. The transport study illustrated that cASP could penetrate through the epithelial monolayer and mainly mediated by the same routes as that in the endocytosis experiment. Moreover, both *in vitro* Ussing chamber and *in vivo* ligated intestinal loops models indicated that cASP could be diffused through the mucus barriers and be absorbed in the whole small intestine. Finally, near-infrared fluorescence imaging presented that cASP could be absorbed and circulated into the blood, then distributed into various organs after oral administration. In conclusion, ASP could be absorbed after oral administration through endocytosis process mainly mediated by macropinocytosis pathway and clathrin- and caveolae (or lipid raft)-related routes, then be absorbed and circulated into blood. This study presents a comprehensive understanding of oral delivery of cASP, which will provide theoretical basis for the clinical application of ASP.

## 1. Introduction

The root of *Angelica sinensis* (Oliv.) Diels (Chinese Danggui) has been traditionally used in Chinese medicinal formulation for a long time, serving as an important blood supplement agent for gynecological diseases in Asia (Dog, 2005). It has also been widely known as a dietary supplement in Europe and America (Deng et al., 2006). In recent years, countless active components have been separated from the root of *A. sinensis* for their various pharmacological activities (Chao & Lin, 2011). Among them, polysaccharides, a major ingredient, have gained remarkable attention due to its exquisite pharmacological effects and low toxic (Liu et al., 2010). Multiple polysaccharides have been extracted from the root of *A. sinensis*. Cao et al. gained an arabinoglucan, which exhibited significant anti-tumor activity both *in vitro* and *in vivo* (Cao et al., 2006). Sun et al. isolated a pectic polysaccharide and presented potential radio protective effect (Sun et al., 2010).

In our previous studies, an acid homogenous heteropolysaccharide, named as ASP, with a molecular weight of approximately 79 kDa, was obtained from the root of *A. sinensis* and the primary structure of ASP was proposed (Zhang et al., 2016). In addition, studies on the pharmacological activity of ASP were also carried out. Previous reports have shown that ASP could promote erythropoietin secretion and reduce hepcidin levels to achieve the antianemic effect (Zhang et al., 2014) and reduce blood glucose levels and ameliorated insulin resistance in high-fat-diet mice (Wang et al., 2016). Both of the pharmacological activities mentioned above were effective through oral administration of ASP and the relevant mechanisms were also described. However, the potential mechanism related to its absorption and tissues distribution in experimental animals after oral administration of ASP is still unclear.

Before reaching the systemic compartment, macromolecule compounds need to overcome multiple barriers. It has been widely recognized that the passage through intestinal epithelium is the major barrier for the oral absorption of macromolecules, which occurs by either paracellular or transcellular pathways (Salama et al., 2006). Paracellular or transcellular pathways are two primary pathways for macromolecules to across the intestinal barriers (Hwang & Byun, 2014; Lundquist & Artursson, 2016). On one hand, paracellular pathway requires the opening of tight junctions to allow the molecule to pass through the gaps between the epithelial cells (Swaan, 1998; Luo et al., 2016). The tight junctions control diffusion of solutes through the paracellular route, allowing passage of certain small hydrophilic molecules but restricting the diffusion of higher molecular weight compounds (Fu et al., 2015). On the other hand, the transcellular pathway involves the passage of the molecules across the apical cell membrane, through the cytoplasm, and across the basolateral membrane by passive diffusion or by a carrier vesicle-mediated process (Riezman et al., 1997). Endocytosis, which transport extracellular polysaccharide into the cell interior, is the initiate procedure of the transcellular pathway (Sahay et al., 2010). Potential mechanism of endocytosis of macromolecules has been relatively well studied. However, as far as we know, limited information was available to judge if polysaccharides, especially with a high molecular weight, could across the intestinal epithelium by either paracellular pathway or transcellular pathway and be absorbed into systemic circulation via the oral delivery.

Due to the lack of sensitive quantitative method, absorption of polysaccharides *in vivo* as well as *in vitro* was rarely investigated. Researchers were increasingly inclined to use the tagging technology to study absorption and tissues distribution of polysaccharide for its rapid expansion in recent years. For instance, Zuo et al. used the fluorescence FITC labeled squid ink polysaccharide to trace its transportation through the Caco-2 cells monolayer (Zuo et al., 2016). Chen et al. reported that ^99m^Tc-labeled H-1-2 (*Pseudostellaria heterophylla* polysaccharide) was used for SPECT/CT studies to investigate the distribution of H-1-2 in mice after oral administration (Chen et al., 2016). Lee et al. employed a near infrared fluorescent dye Cy5.5 labeled ZnO nanoparticles for near-infrared fluorescence imaging to investigate the behavior and biodistribution of the marker in mice after oral exposure (Lee et al., 2012). Obviously, radioactive compound is a potential health hazard and environmentally unfriendly. Synthesis and disposal of radioactive compound are expensive. For some isotopes with relatively short half-lives, radioactivity decay is quite rapid and thus, the compounds lose their usefulness in time (Liu et al., 2012). Fluorescent dyes conjugated the drug to produce optical probes used *in vivo* and *ex vivo*. This technology is relatively safe, low cost, and noninvasive (Dong et al., 2017). However, compared with none-infrared fluorescent dyes, infrared fluorescent dyes correspond to low photon absorption and auto fluorescence in tissues, which not only can obtain effective imaging of macromolecules both *in vitro* and *in vivo*, especially in deep tissues, but also can track and monitor macromolecules in the body with higher sensitivity and specificity (Mizukami, 2017). In view of both the advantages and disadvantages of these methods, the near infrared fluorescence labeling method was finally selected to label ASP.

In the present study, ASP was conjugated with a near infrared fluorescent dye Cy5.5 as a fluorescence marker to evaluate the oral absorption of ASP. Caco-2 cell monolayer model was selected to form simulative intestinal epithelial cell monolayer for the transport mechanism *in vitro* (Domínguez-Avila et al., 2017). The Ussing chamber and ligated intestinal loops models, frequently used to study the permeability and absorption kinetics of drugs (Billat et al., 2017), were elected to evaluate the permeability of ASP in different parts of the intestine in this study. Finally, near-infrared fluorescence imaging was used to investigate whether ASP could be absorbed by gastrointestinal (GI) tract and circulated into the blood.

## 2. Materials and methods

### 2.1. Materials

*Angelica sinensis* polysaccharide was obtained and characterized according to our previous studies. 1-Ethyl-3-(3-dimethylaminopropyl) carbodiimide hydrochloride (EDC·HCl), hydroxysuccinimide (NHS), succinic anhydride (SA), and Cy5.5 azide (Cy5.5-N_3_) were purchased from Lumiprobe Corporation (Hallandale Beach, FL). Sodium azide, nystatin, chlorpromazine, cytochalasin D, and 5-(N-ethyl-N-isopropyl) amiloride (EIPA) were obtained from Sigma-Aldrich (St. Louis, MO). Caco-2 cell line was purchased from the Shanghai Cell Bank of Chinese Academy of Sciences. Dulbecco’s modified Eagle’s medium (DMEM, high-glucose), fetal bovine serum (FBS), nonessential amino acids, penicillin, streptomycin, and 0.25% trypsin-EDTA solution were obtained from Gibco BRL (Gaithersburg, MD). All other chemical reagents were analytical reagent grade.

### 2.2. Synthesis and characterizations of cASP

#### 2.2.1. Preparation of ASP-SA

After 60 mg ASP was dissolved in DMSO solution, 10 mg of SA and 20 mg of DAMP were added. Then the solution was kept stirring for about 24 h at 50 °C. The solution was dialyzed against distilled water for 3 days and lyophilized to get ASP-SA.

#### 2.2.2. Preparation of Cy5.5-ASP

40 mg ASP-SA was dispersed in 10 mL PBS (pH 5.0), followed by the addition of 50 mg of EDC and 32 mg of NHS for activation. After that, 1 mL of Cy5.5 azide was added (2 mg dissolved in DMF solution) and the pH was adjusted to 8.0. The reaction was allowed to proceed under stirring for 48 h at room temperature. Then, the product was purified by dialysis against deionized water in a cellulose dialysis bag (MWCO = 3500 Da), followed by lyophilization to obtain Cy5.5-ASP, named as cASP.

#### 2.2.3. Characterizations of cASP

IR spectra of ASP, ASP-SA, and cASP were recorded with FT- IR spectrometer (Bruker Vertex 70; Karlsruhe, Baden-Württemberg, Germany) in the region of 4000–400 cm^−1^ for detecting functional groups. ^1^H NMR spectra was recorded with a Bruker AVIII-600 NMR spectrometer (Bruker Corporation; Karlsruhe, Baden-Württemberg, Germany). 10 mg ASP, 10 mg ASP-SA, 10 mg cASP, and 10 mg SA were dissolved in 99.9% D_2_O, and then lyophilized. The operation described above would be done in triplicate to ensure complete heavy water exchange in the samples. Thereafter, the samples were dissolved in 0.5 mL of 99.9% D_2_O, respectively. Meanwhile, 10 mg Cy5.5 was dissolved in 99.9% DMSO-d_6_ (Cy5.5 was insoluble in D_2_O) for ^1^H NMR spectra analysis.

### 2.3. Near-infrared fluorescence (NIRF) imaging

All procedures involving animals were conducted in strict accordance with the Guidelines of the Institutional Animal Care and Use Committee of Tongji Medical College and the National Institutes of Health Guide for the Care and Use of Laboratory Animals (permit number: SYXK (HuBei) 2015-0018), Tongji Medical College, Huazhong University of Science and Technology. Twenty male BALB/c mice (20 ± 2 g) maintained in polypropylene cages (five in each cage) in an air-conditioned room (25 ± 1 °C, relative humidity 50 ± 20%,12-h light/dark cycle). A 4 mg of cASP in 0.5 mL distilled water was orally administrated to the mice. Optical images for cASP were acquired using the *In vivo* FX PRO imaging system (Bruker Corporation, Germany). *In vivo* near-infrared fluorescence imaging was performed at 1, 2, 4, 6, and 8 h after oral administration. In the study of *ex vivo* imaging, the mice were divided into groups (*n* = 3) for different time point analysis. The mice were sacrificed and their major organs, that is, the heart, lung, liver, spleen, kidney, stomach, and small intestine were collected and imaged at the same time points.

### 2.4. In vitro intestinal absorption of cASP by Ussing chamber model

Six male SD rats (250 ± 20 g body weight) were fasted overnight, but supplied with water *ab libitum* before the experiment. For preparation of the tissue segments, the rats were anesthetized with urethane and the intestinal segments (duodenum, jejunum, ileum) were immediately removed and placed in cold (4 °C), bubbled (O_2_–CO_2_ 95: 5) Krebs' Ringer bicarbonate buffer (KRB). Each section of the small intestine was washed and cut with opened along the mesenteric border, which was mounted in Ussing chamber with an exposed tissue area of 1.78 cm^2^. Areas with evident Peyer’s patches were not used for the experiments. Each of the half-cells (i.e. mucosal and serosal sides) in the Ussing chamber was filled with 4 mL KRB and equilibrated for 30 min to regain physiological stability before commencing permeability experiment. After equilibration, the KRB was removed and replaced with pre-warmed (37 °C) cASP solution (100 μg/mL) in KRB in the mucosal side and 4 mL of fresh buffer was added to the serosal side. Then, 0.5 mL samples were withdrawn from the serosal side at different time intervals and replaced with fresh KRB solution. The temperature of the chamber was maintained at 37 °C in whole experiment. Three independent experiments were performed for each segment.

The *Q* (accumulative quantity) and *P*_app_ (apparent permeability) across the excited rat intestinal segment in the Ussing chamber were calculated using the following equations:

(1) $$Q=\text{4}C_{n}+\;0.\text{5 }\times \sum_{i=1}^{n-1}C_{i}$$

(2) $$P_{\text{app}}=\left(\text{d}Q/\text{d}t\right)/\left(A\times C_{0}\right)$$

### 2.5. Ligated intestinal loops model *in vivo*

Nine male SD rats were weighted and grouped (*n* = 3) to ensure that no considerable difference in weight appeared between groups. The rats were anesthetized with urethane, and then 2 cm section from small intestinal loops were obtained and washed with KRB solution (37 °C). cASP dissolved in KRB solution (100 μg/mL, 0.5 mL) was injected into each loop and then ligated at both ends. After 2 h, the rats were sacrificed, and the section of each loop was removed and washed with KRB solution to clean the residual cASP. Subsequently, the small intestinal tissues were frozen sections and stained. Finally, the tissue sections were visualized with confocal laser spectrum microscope (LSM 780, Zeiss Company, Oberkochen, Germany).

### 2.6. Caco-2 cells culture

Caco-2 cells (human intestinal epithelial cell) were cultured with DMEM supplemented with 10% (v/v) FBS (fetal bovine serum), penicillin and streptomycin (100 U/mL), and 1% nonessential amino acids with the environmental condition maintained at 37 °C in an atmosphere of 5% CO_2_. Caco-2 cells were seeded into 12 well polycarbonate transwell plates at a density of 1 × 10^5^ cells and grown for 21 days in order to form a confluent monolayer (pore size: 0.4 μm, surface area: 1.12 cm^2^). A 0.5 mL of DMEM containing 1 × 10^5^ cells was seeded in the upper compartment, and 1.5 mL of cell-free DMEM was poured into the lower compartment. Culture medium was changed every 2 days in the initial 2 weeks and then every day in the following week. The integrality of the Caco-2 cell monolayer was verified by measuring the TEER value using a Millicell-ERS volt-ohmmeter (Millipore Company, Bedford, MA). The TEER value of the Caco-2 cell monolayer exceeding 400 Ω/cm^2^ was considered to be qualified for subsequent experiments.

### 2.7. Cytotoxicity of the ASP in the caco-2 cells line

#### 2.7.1. Cell viability assays

The cytotoxicity test of the cASP against Caco-2 cells was performed by MTT assay. Briefly, 1.0 × 10^4^ cells per well were seeded in a 96-well plate. After the cells were approximately 80% confluent, the cells were exposed to a series of concentrations of the cASP (50–800 μg/mL) at 37 °C for 24 h. After that, the cells were incubated with 20 μL of MTT solution (5 mg/mL) per well for another 4 h. Then the supernatants were discarded, and 100 μL of DMSO was added to dissolve the insoluble formazan-containing crystals. Finally, the plate was shaken for 30 min at 37 °C, and the absorbance of each well was measured at 490 nm by a microplate reader.

#### 2.7.2. Lactate dehydrogenase (LDH) release assay

The effect of cASP transport on the cell membrane was detected using a lactate dehydrogenase assay kit (Beyotime, Haimen, Jiangsu, China). Briefly, the Caco-2 cells cultured in a 96-well plate were incubated with serum-free DMEM containing various concentrations of cASP at 37 °C for 4 h. In addition, cells incubated with serum-free DMEM alone were used as a control group. One hour before the detection, 20 μL of LDH release solution was added to the positive control group (without drug-treated cell wells for subsequent cell lysis). Then, 120 μL of the cells supernatant was transferred to another 96-well plate (Qiao et al., 2017). After being treated according to the manufacturer’s instructions, the absorbance of the supernatant was measured at 490 nm using an automatic microplate reader.

### 2.8. Cellular uptake of ASP

#### 2.8.1. Flow cytometry method (FCM)

The internalization of ASP into Caco-2 cells was investigated by a LSRII flow cytometry (Becton, Dickinson and Company, Franklin Lakes, NJ). Caco-2 cells were grown in 12-well plates for 24 h, and then cASP (100 μg/mL) was added into plates and incubated with cells for varying time intervals. After incubation, the cells were washed with cold PBS three times. Then, the cells were digested and collected for the FCM analysis. Moreover, to investigate whether the internalization process was energy-dependent, Caco-2 cells were treated with low temperature (4 °C) or sodium azide (1 mg/mL) for 1 h and then incubated with cASP (100 μg/mL) for another 2 h. Then, the cells were collected for detection by FCM.

#### 2.8.2. Confocal laser spectrum microscope method (CLSM)

After Caco-2 cells were seeded onto glass slides, 100 μg/mL cASP was added into plates and incubated with cells for 0.5, 2, and 4 h. After incubation, the cells were washed three times with cooled PBS and fixed by 4% formaldehyde for 15 min. Finally, cell nuclei were stained for 15 min with DAPI. The samples were observed with CLSM. As to the energy inhibition process, according to the operation mentioned above, then fixed and stained for CLSM.

#### 2.8.3. Investigation of endocytosis pathways in Caco-2 cells

Firstly, the Caco-2 cells were pretreated with different transport inhibitors for 1 h: (I) 30 μM of nystatin, (II) 30 μM of chlorpromazine, (III) 100 μM of EIPA, (IV) 5 μM of Cytochalasin D. Then, the cells were incubated with 100 μg/mL of cASP containing the same inhibitors for 2 h. Subsequently, the Caco-2 cells were washed three times with cold PBS and analyzed by CLSM and FCM as described above.

### 2.9. Transport of the cASP across Caco-2 cells monolayer

#### 2.9.1. cASP transport through Caco-2 cells monolayer

The monolayer was washed three times with HBSS, then incubated with HBSS in CO_2_ incubator for 30 min at 37 °C for equilibrating. In the transport study, 0.5 mL of cASP (100 μg/mL) HBSS solution was added to the apical side followed by addition of 1.5 mL of HBSS solution to the basolateral side for transportation. At certain time intervals, 500 μL solution was removed from basolateral side and rapidly replaced with equivalent fresh HBSS solution. The content of cASP was detected by fluorescence spectrometer. During the experiment, the integrity of the monolayer was assessed by TEER value measurement. The apparent permeability coefficient (*P*_app_) was calculated to evaluate the transportation ability of cASP.

#### 2.9.2. Investigation of endocytosis pathways in the Caco-2 cells monolayer

The Caco-2 cells monolayers were pretreated with different endocytosis inhibitors mentioned above for 1 h. Then, the Caco-2 cells monolayers were incubated with 100 μg/mL of cASP containing the corresponding transport inhibitors for another 2 h. At the end of the incubation, the amount of cASP transported to the basolateral side (in the lower side) was measured by a fluorescence spectrophotometer.

### 2.10. Statistics analysis

All data were presented as mean ± SD for three replicates for each prepared sample. The statistical difference between the groups was analyzed using analysis of variance (ANOVA) and considered significant with *p* < 0.05. The corresponding markers in figures were defined as *p < 0.05 and **p < 0.01, respectively.

## 3. Results and discussion

### 3.1. Characterizations of cASP

**Figure 1.**
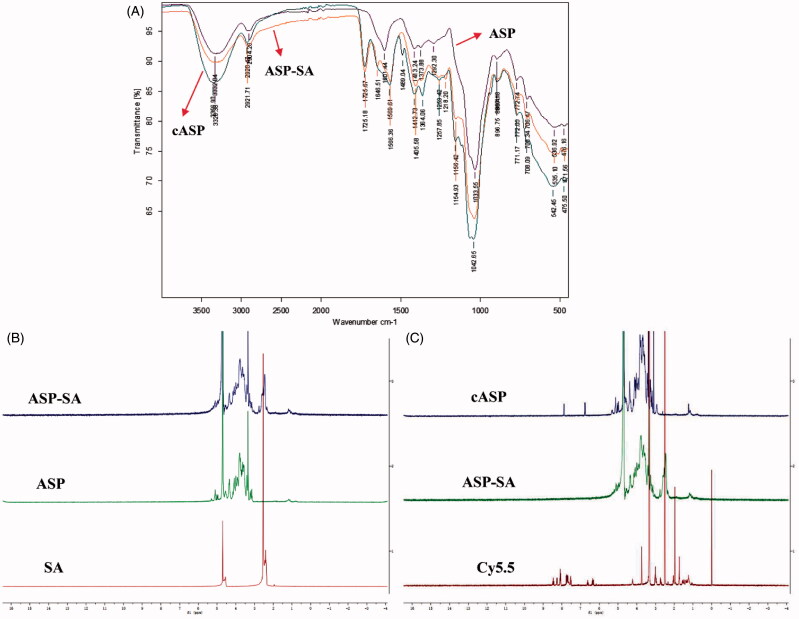
Characterizations of ASP, ASP-SA, and cASP. (A) FT-IR spectra in the range of 4000–400 cm^−1^. (B) ^1^H NMR spectra of SA, ASP, ASP-SA. (C) ^1^H NMR spectra of Cy5.5, ASP-SA, cASP.

In this study, ASP was chemically modified and conjugated with Cy5.5 and the characterizations of cASP were investigated. Figure 1(A) shows the infrared spectra of ASP, ASP-SA, and cASP. Compared with the IR spectrum of ASP, which presents a typical polysaccharide infrared spectrum (Lai et al., 2017), the IR spectrum of cASP presented some extra peaks. Those peaks were observed at 3328 cm^−1^ (vibration of –OH, –NH_2_), 1725 cm^−1^ (vibration of –O–C = O), 1646 cm^−1^ (secondary amide I band: stretching vibration of –C = O), 1566 cm^−1^ (secondary amide II band: deformation vibration of –NH and stretching vibration of –C–NH). ^1^H NMR spectrum is shown in Figure 1(B, C). A 4.7 ppm and 2.5 ppm were assigned as the solvent peaks of D_2_O and DMSO-d_6_, respectively. The resonance signals in the ranges of 3.0–5.5 and 2.3–2.6 ppm were assigned as the protons in sugar units of ASP and methylene protons of SA residues (Jiang et al., 2007; Zhang et al., 2016). In the ^1^H NMR spectrum of the ASP-SA (Figure 1(B)), the resonance signals of the protons of ASP (3.0–5.5 ppm) were also observed and a multiplet was present at 2.3–2.6 ppm region, corresponding to the methylene proton of SA residues. The result confirmed that the SA residues conjugated with the ASP. Compared with the ^1^H NMR spectra of ASP-SA, Cy5.5, and cASP, the characteristic resonance signals of aromatic groups of Cy5.5 (6.0–8.5 ppm) and protons of ASP (3.0–5.5 ppm) were observed in the ^1^H NMR spectrum of the cASP derivative (Figure 1(C)). It should be noted that the characteristic resonance peaks of SA protons were observed in the ^1^H NMR spectrum of the ASP-SA derivative, which were absent in the ^1^H NMR spectrum of the cASP. These results evidenced that Cy5.5 residues were conjugated with cASP successfully.

### 3.2. Near-infrared fluorescence imaging

**Figure 2.**
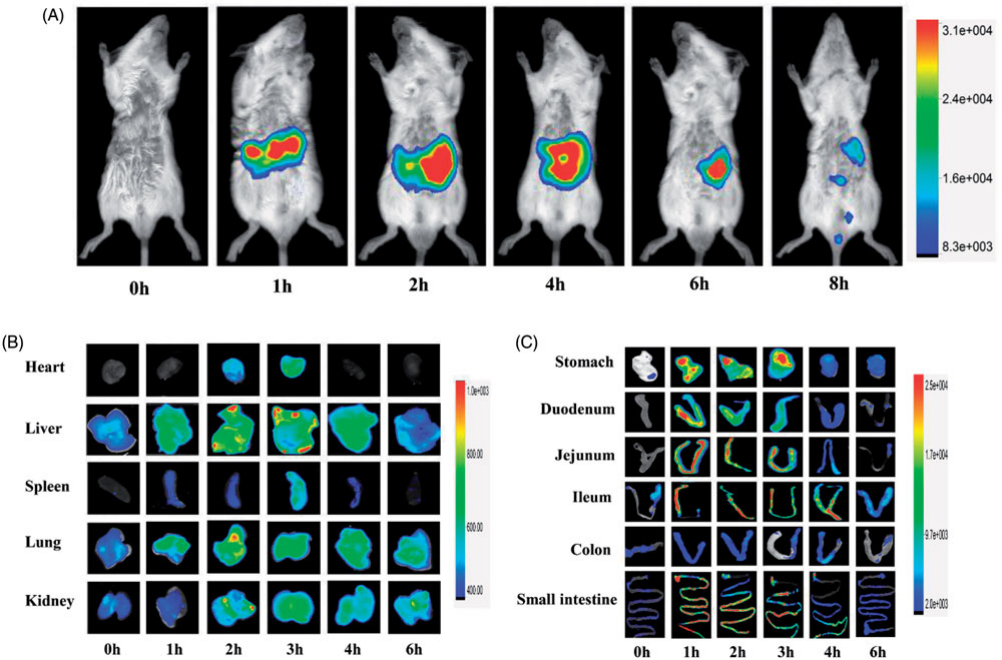
*In vivo* biodistribution profile of cASP orally administered, in healthy mice. (A) Fluorescence intensity images of mice whole body from 0 to 8 h. (B) *Ex vivo* imaging of major organs (heart, liver, spleen, lung, kidney) from 0 to 6 h. (C) *Ex vivo* imaging of gastrointestinal tract from 0 to 6 h.

The *in vivo* behavior of ASP was evaluated by applying it for *in vivo* imaging. NIRF imaging, as a complement to nuclear imaging methods, is a powerful tool for intravital imaging (Yan et al., 2015). It provides both anatomical and functional/molecular information by using exogenous fluorescent probes. Cy5.5 is broadly used in NIRF imaging studies because of its regnant characteristics, including relative bulkiness, small Stokes shift, low signal loss, and strong anti-jamming capability (Licha et al., 2000). In our present study, ASP was labeled with Cy5.5 and be applied to NIRF imaging to get a visual biodistribution and absorption of ASP in the body. Figure 2(A) shows the whole mice body images after oral administration of cASP. The signal intensity was mainly observed in the GI tract, indicated that a considerable portion of cASP reached the GI tract. This finding was consisted with the result of Chen et al. (2016), whom studied the biodistribution of ^99m^Tc-labeled *Pseudostellaria heterophylla* polysaccharide. It was noteworthy that signal intensity did not be found in the lung in the whole mice body imaging, which was the main distribution organ after oral administration of free Cy5.5 (Hue et al., 2013), indicated that Cy5.5 dissociate from the ASP during absorption and biodistribution after oral administration. Then, to identify relatively weak fluorescent signal in other organs (e.g. heart, liver, spleen, lung, kidney), the *ex vivo* optical imaging studies were investigated. The result presented that their signal intensity increased when time was prolonged (Figure 2(B)), indicating that orally administered cASP could be absorbed and circulated into the blood, then distributed into various organs. In addition, it was observed a relatively higher accumulation of cASP in the liver and kidney than in other organs. This phenomenon might be related to the structure of ASP. Previous report has been indicated that ASP mainly consisted of galactose (Zhang et al., 2016), then ASP could bind to the asialoglycoprotein receptor (Pranatharthiharan et al., 2017) in the liver surface, leading to the relatively strong signal intensity in the liver. As to the high intensity in kidney, it was speculated that cASP might be clear from the body via the kidney. Moreover, we performed *ex vivo* optical imaging with the GI tract to confirm the specific absorption segment of cASP. As shown in Figure 2(C), the whole GI tract had a strong signal intensity, which demonstrated that cASP could be absorbed in the whole small intestine. It was a remarkable fact that the absorption mainly occurred in the jejunum and ileum of the small intestine, especially in the ileum for its more notable absorption than other sections. However, the absorption of cASP did not occurred in the colon. To our knowledge, owing to the severe GI environment, a significant proportion of polysaccharides were fermented by bacterial enzymes and disintegrated into smaller units (Flint et al., 2008; Reintjes et al., 2017). Chondroitin sulfate, which was a glycosaminoglycan, was degraded into oligosaccharides by bacteria in the GI tract and eventually be absorbed (Barthe et al., 2004). However, in order to verify the metabolic status of cASP in the body, both *in vitro* and *in vivo* digestion assay of cASP was provided. It was found that the ASP in the serum was intact molecules and not broken into smaller fragments (Supplementary Figure S1). This result supported the view that polysaccharides might be absorbed in the form of macromolecules (Cai et al., 2017). Therefore, it could be confirmed that a certain amount of cASP was absorbed in small intestine and enter the systemic circulation the form of macromolecule, in despite of the considerable barriers in the GI tract.

### 3.3. *In vitro* intestinal absorption of cASP by Ussing chamber model

**Figure 3.**
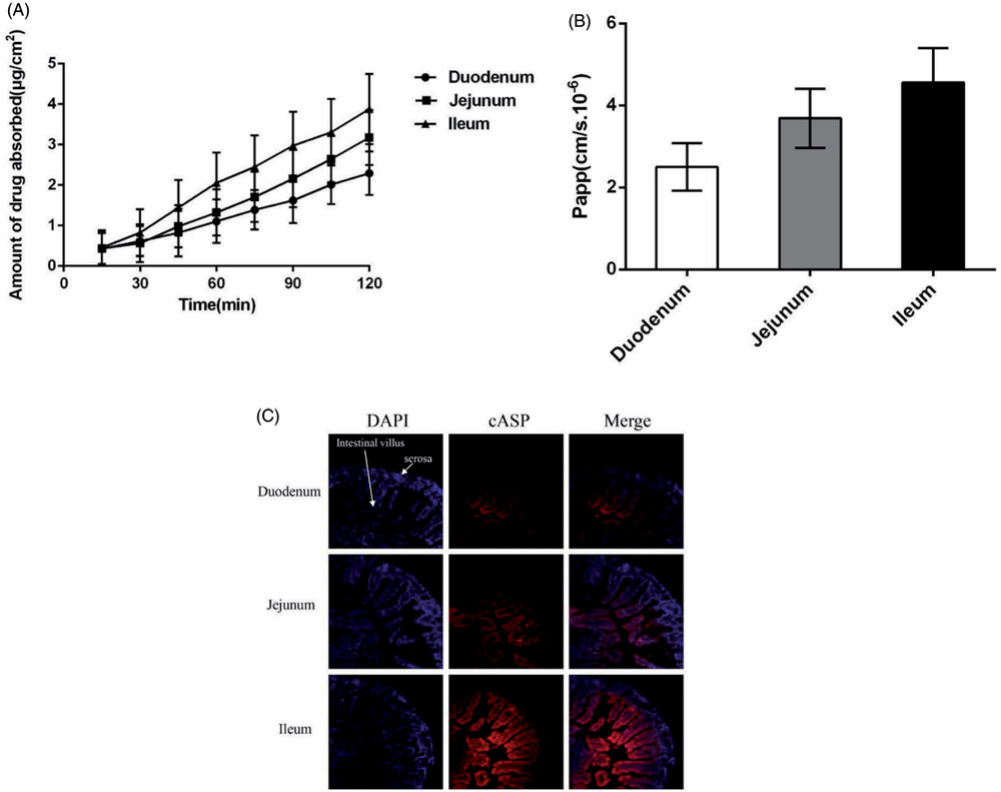
Absorptive characteristics of cASP in the Ussing chamber model *in vitro* and ligated intestinal loops *in vivo* from three different intestines. (A) The accumulated transport amount curve. (B) The *P*_app_ value from three different intestines. (C) Distribution of cASP in small intestine villi with ligated intestinal loops. Among which, blue fluorescence represent the nucleus of small intestinal cells and red fluorescence refer to cASP. All of the data represent the mean ± SD (*n* = 3).

The intestinal absorptive behavior of cASP in different intestinal segments (duodenum, jejunum, ileum) was studied by the Ussing chamber technique. The concentration of cASP measured by fluorescence spectrometry quantification analysis at *λ*_ex_ = 670 nm and *λ*_em_= 710 nm, and then the accumulated transport amount and the apparent permeability coefficient (*P*_app_) of cASP were calculated. The results present in Figure 3 indicated that the accumulated transport amount of cASP was time-dependent. The *P*_app_ value of cASP in three section intestines were about 2.51 × 10^−6 ^cm/s, 3.69 × 10^−6 ^cm/s, and 4.56 × 10^−6 ^cm/s, respectively. The ileum showed the highest *P*_app_ value, which coincided well with the results of near-infrared fluorescence imaging. The different absorption profile in small intestinal segments might ascribed to the functional and structural differences between duodenum, jejunum, and ileum. Duodenum could be primarily responsible for the chemical digestion of food using enzymes instead of absorption and jejunum primarily absorbed the nutrients and smaller molecules by specialized villi. However, ileal bile acid transport (IBAT) was highly expressed in the ileum (Sokolis, 2017), which might increase the absorption of cASP. Previous studies demonstrated that the absorption of chitosan, which was a well-known and FDA-approved candidate biomaterial carrier for many drugs, was mainly occurred in duodenum and jejunum (Chae et al., 2005). Different absorption regions might be implied different mechanism between chitosan and cASP. Electrical resistance across the intestinal epithelia increased from the duodenum to the ileum indicating increased resistance to passive ion flow in the distal regions of the small intestine (Loehry et al., 1973). Chitosan has been proved that mainly be transported by passive diffusion through small intestinal (Wu et al., 2014), nonetheless, result of the present study suggested that cASP absorption in the GI tract did not be ascribed to the simple passive diffusion.

### 3.4. Ligated intestinal loops model *in vivo*

To get better understanding of the permeation of cASP in villi, the nuclei were stained using DAPI and appeared blue, while the red fluorescence corresponded to cASP. As shown in Figure 3(C), the duodenum and jejunum exhibited relatively weak red fluorescence and cASP mainly adhered to the surface of intestinal villus, which might be strongly trapped by highly viscoelastic mucus barriers of the GI tract (Tang et al., 2009). However, unlike duodenum and jejunum, a certain amount of cASP could penetrate through the mucus layer and reach the basolateral epithelial membrane in the ileum. It was deduced that the absorption of cASP in the ileum was higher than that in other two segments, which was consistent with the results of near-infrared fluorescence imaging and Ussing chamber model. On its way through the GI tract, there is a general consensus that any macromolecule would encounter a series of barriers before it reached the capillaries in the subepithelial tissue, including mucus barrier, epithelial cells, basolateral membrane barrier, and so on (Lundquist & Artursson, 2016). Numerous interactions take place in the GI tract that directly impact the absorption feature of macromolecules (Giacco et al., 2016). A great deal of studies have indicated that after being injected into loop, drugs might appear in: (1) remaining in the intestinal lumen, (2) adhering to mucin network and trapped in mucus barrier, (3) penetrating through the mucus layer for possible entry to the basolateral epithelial membrane (Galindo-Rodriguez et al., 2005; Tang et al., 2009; Maisel et al., 2015). In the present study, cASP could penetrate through the mucus layer, enter to the basolateral epithelial membrane, and then be absorbed into systemic circulation.

### 3.5. Cytotoxicology of the cASP in the Caco-2 cells line

**Figure 4.**
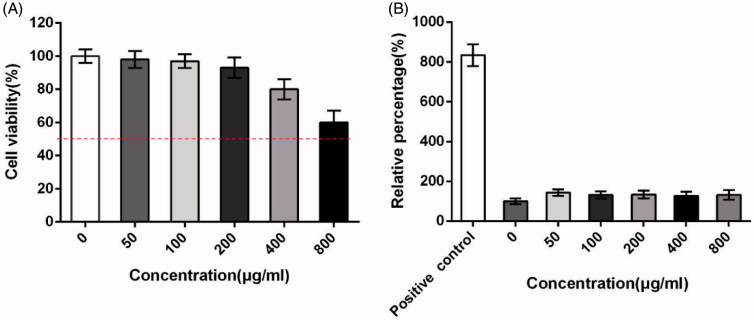
Cytotoxicology of cASP in the Caco-2 cells line. (A) Cytotoxicity tests performed by MTT assay. (B) The integrity of cell membrane detected by lactate dehydrogenase (LDH) release assay. Each data represents the mean value of three tests.

Figure 4(A) shows the cytotoxic effect of the ASP against Caco-2 cells by a MTT assay. The results indicated that cASP did not significantly influence the viability of Caco-2 cells in 24 h, which stated that it could be used for the following experiments.

The effect of the cASP on cell membrane integrity evaluated by a lactate dehydrogenase (LDH) release assay is shown in Figure 4(B). LDH is the cytosolic enzyme and its presence in the luminal fluid is generally regarded as evidence of cell membrane damage (Surampalli et al., 2016). From the result it was found that LDH release of all groups did not show significant discrepancy at the tested concentrations (50 − 800 μg/mL) against the negative control group. It was indicated that no effect of cASP on the integrity of cell membrane. Therefore, it was presumed that cASP diffused into Caco-2 cells in the following experiments could not be attributed to disrupt cell membrane integrity.

### 3.6. Transportation of cASP in Caco-2 cells

**Figure 5.**
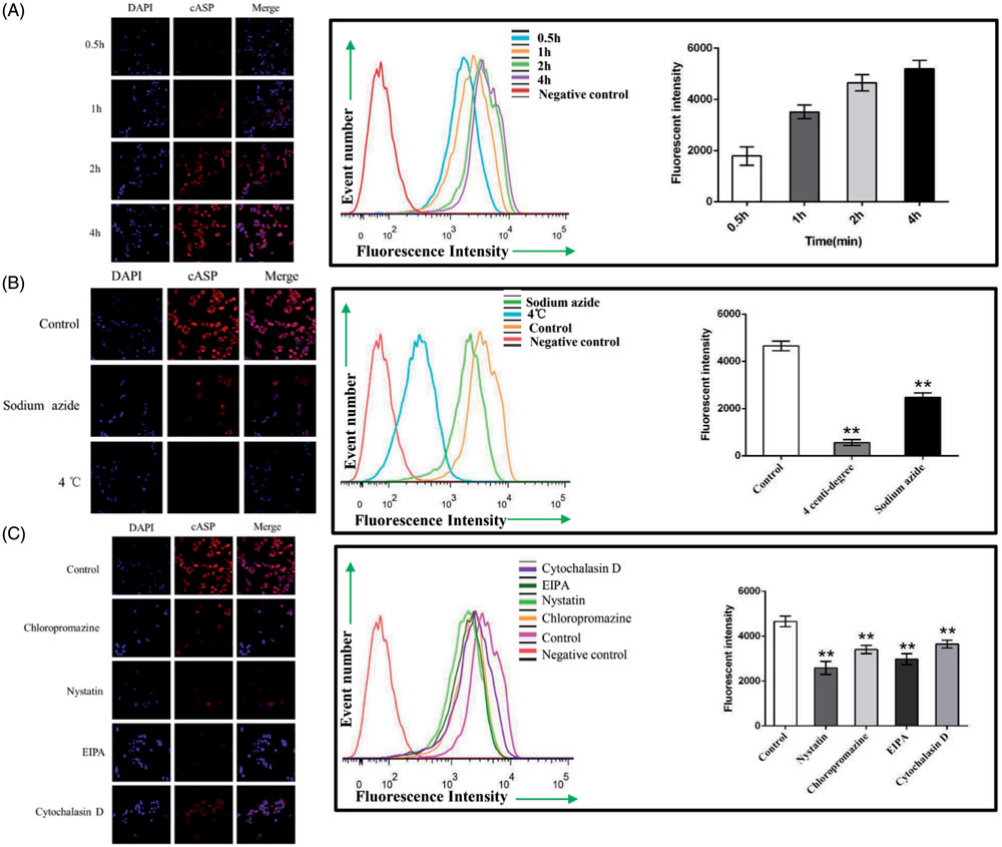
Quantitative or qualitative analysis of the internalized cASP in Caco-2 cells using flow cytometry (FCM) and confocal laser spectrum microscope method (CLSM), respectively. (A) The effect of incubation time on the internalization of cASP. (B) Impacts of sodium azide or 4 °C on the internalization of cASP. (C) Impacts of various transport inhibitors on the internalization of cASP. All of the data represent the mean ± SD (*n* = 3) and **p < .01 versus control.

The transport of cASP in Caco-2 cells was investigated by quantitative FCM and qualitative CLSM. As shown in Figure 5(A), the cellular uptake of cASP increased remarkably when the incubation time was prolonged. To further determine whether the uptake of cASP was an energy-dependent process, Caco-2 cells were treated with 4 °C or sodium azide, which could effectively inhibit the active transport process. Figure 5(B) shows that the cellular uptake dramatically decreased. Previous studies have indicated that macromolecules with MW > 700 permeated cell membrane via active transport rather than passive transport (Hwang & Byun, 2014). Iwasa et al. also proved that endocytosis was an energy-dependent process because the formation and transport of the vesicles were driven by coupling with ATP hydrolysis (Iwasa et al., 2006). Our study was consistent well with these previous studies and indicated that the cellular uptake of cASP was both time- and energy-dependent.

### 3.7. Investigation of endocytosis pathways in Caco-2 cells

Plenty of evidences have shown that several pathways for the endocytosis of extracellular macromolecules into cells, including the macropinocytosis pathway, clathrin- and caveolae (or lipid raft)-related routes (Swaan, 1998; Sahay et al., 2010). Meanwhile, actin filaments were known to be critical components in the formation of plasma-membrane invaginations and their scission, which played a prominent role in the internalization and transportation of macromolecules (Ben-Dov & Korenstein, 2013). Chloropromazine, which blocks the assembling of clathrin at the cell membrane, is typically used to inhibit the clathrin-mediated route (Kadlecova et al., 2016). Nystatin, which is a cholesterol sequestering agent, is known to function as an inhibitor of caveolae (or lipid raft) mediated endocytosis (Cartwright et al., 2012). EIPA has been reported to effectually inhibit the macropinocytosis-mediated route (Devadas et al., 2014). Cytochalasin D can disrupt actin filaments (Conner & Schmid, 2003). After intervention with these inhibitors, the results are shown in Figure 5(C). Compared with the cellular uptake in the control group, a 49.0% reduction of cASP in the presence of nystatin indicated that cellular uptake of cASP mainly mediated by caveolae (or lipid raft)-related pathway. A 36.0% reduction was also found in the presence of EIPA, which indicated that macropinocytosis route also played a significant role in the internalization of cASP. However, cASP internalization under conditions of chloropromazine and cytochalasin D decreased slightly, suggesting that clathrin-related and actin filaments played relatively minor roles in the endocytosis process.

### 3.8. Transport of cASP in Caco-2 cells monolayer

#### 3.8.1. cASP transport through Caco-2 cells monolayer

Caco-2 cells can form confluent monolayer with the functional properties of intestinal epithelium, which are widely used for studying drug transport mechanism. Accordingly, the permeability coefficient evaluated using in Caco-2 cells monolayer experiment is regarded as a reference for intestinal drug permeability (Billat et al., 2017).

**Figure 6.**
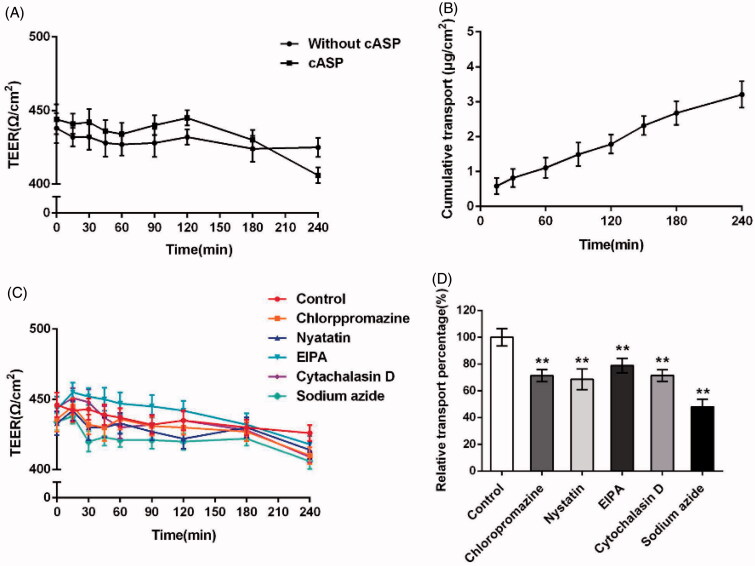
Evaluation of the transport of cASP across Caco-2 cells monolayer. (A) TEER change of Caco-2 monolayers when incubating with cASP and fresh HBSS solution. (B) The cumulative amount of cASP in the basolateral chamber as the function of time. (C) TEER change of Caco-2 monolayers when incubating with different endocytosis inhibitors, fresh HBSS solution as a control group. (D) The effects of different endocytosis inhibitors on the transport of cASP across Caco-2 cells monolayer. All of the data represent the mean ± SD (*n* = 3) and **p < .01 versus control.

To address whether cASP could be transported through epithelial cells, the TEER at various time points within 4 h and the amount of cASP transported across the Caco-2 cells monolayer were detected. Duizer et al. have demonstrated a good correlation between TEER values and the monolayer integrity (Duizer et al., 1999). Transportation of macromolecule in Caco-2 cells monolayer could be mediated by either transceclular or paracellular pathways as reported. Paracellular pathway required the opening of tight junctions to allow the molecule to pass through the gaps between the epithelial cells, with the result of reducing TEER value (Kowapradit et al., 2010; Fu et al., 2015). The permeation of high weight polysaccharides through the paracellular pathway was previously shown to be restricted by tight junctions (Artursson et al., 2001). In our study, neither without cASP group nor cASP group displayed a discernable drop in TEER at any time point within 4 h (Figure 6(A)), which indicated that cASP was rarely transported across the Caco-2 cells monolayer by paracellular pathway. The accumulated transport amount of cASP increased during the 4 h incubation and showed a relatively good linear relevance with time. At 240 min, the accumulated transport amount was 3.21 μg/cm^2^ (Figure 6(B)). Furthermore, we calculated the permeability coefficient (*P*_app_) of cASP basis on the curve to evaluate its transportation ability. The *P*_app_ value was 1.33 × 10^−6 ^cm/s, which indicated that cASP could be absorbed by the epithelial cells, although it was generally believed that the polysaccharide administrated orally could not be directly absorbed by GI tract into blood stream for functional activities owing to its macromolecule and complex structure (Michell et al., 1996). There have been reported that certain polysaccharides could be transported across the intestinal epithelium. For example, Nagamine et al. reported that high molecular weight fucoidan was absorbed through intestinal epithelial cells both *in vivo* and *in vitro* (Nagamine et al., 2014). Zuo et al. proposed that squid ink polysaccharide could be permeated through the Caco-2 cells monolayer and *P*_app_ was 1.44 × 10^−6 ^cm/s (Zuo et al., 2016). Our results were consistent with the above previous studies and supported the notion that an intestinal uptake of certain polysaccharides occurred.

#### 3.8.2. Investigation of endocytosis pathways of cASP in the Caco-2 cells monolayer

Various inhibitors were used to confirm the mechanism of transport of cASP through Caco-2 cell monolayer. Figure 6(C) presents that inhibitors used in this study exerted little influence on the TEER values of the Caco-2 cell monolayer, suggesting that had no effect on the integrity of Caco-2 cell monolayer. Figure 6(D) illustrates that sodium azide, which inhibited the energy-dependent process, was able to dramatically decrease the transportation of cASP. The finding indicated that the transportation of cASP was energy-dependent. In addition, chloropromazine, nystatin, and EIPA, could decreased the transportation of cASP by 29%, 32%, and 21%, respectively, demonstrating that macropinocytosis pathway and clathrin- and caveolae (or lipid raft)-related routes all participated in the transportation of cASP cross Caco-2 cell monolayer. Furthermore, Cytochalasin D also decreased the transportation of cASP similar to chloropromazine, shedding light on that actin filaments played an important role in the procedure. In the endocytosis study, the addition of EIPA exerted a strong suppression of cASP endocytosis. However, EIPA created a relativity gentle inhibitory effect on the process of transportation through Caco-2 cells monolayer compared with other inhibitors. The reason for the difference might be due to the different activity of cell membrane and cell monolayer. Cell monolayer displayed microvilli and tight junctions, and expressing P-gp and several relevant efflux transporters and enzymes, in which various interactions might influence the macropinocytosis pathway in the transport process through the Caco-2 cells monolayer (During & Harrison, 2005).

## Conclusion

In conclusion, this study presents a comprehensive understanding of oral absorption of ASP, which will provide theoretical basis for the clinical application of ASP. It has been confirmed that ASP could be absorbed after oral administration through endocytosis process mediated by macropinocytosis pathway and clathrin- and caveolae (or lipid raft)-related routes, then be absorbed and circulated into blood. However, the endocellular transport and exocytosis process remains a further systematic study in the future.

## Supplementary Material

IDRD_Zhang_et_al_Supplemental_Content.doc
