# Supplementary material for: Investigation of the transport and absorption of Angelica sinensis polysaccharide through gastrointestinal tract both in vitro and in vivo
Source: Drug Deliv. 2017 Sep 18;24(1):1360–71. doi: 10.1080/10717544.2017.1375576 (PMC8240978; doi:10.1080/10717544.2017.1375576)
Supplement: IDRD_Zhang_et_al_Supplemental_Content.doc [file IDRD_A_1375576_SM6017.doc]

**Investigation of the Transport and Absorption of *Angelica Sinensis* Polysaccharide through Gastrointestinal Tract both *in Vitro* and *in***

***Vivo***

Kaiping Wang^a^, Fang Cheng^a^, Xianglin Pan^a^, Tao Zhou^b^, Xiqiu Liu^a^, Ziming Zheng^b^, Li Luo^b, *^, Yu Zhang^b,*^

^a^ *Hubei Key Laboratory of Nature Medicinal Chemistry and Resource Evaluation, Tongji Medical College, Huazhong University of Science and Technology, 430030, Wuhan, China*

^b^ *Union Hospital of Tongji Medical College, Huazhong University of Science and Technology, No. 1277 Jiefang Road, 430022, Wuhan, China*

*Corresponding author: Tel: +86 27 63559222; Fax: +86 27 63559222

E-mail address: zhangwkp@163.com

**Supplementary Materials**


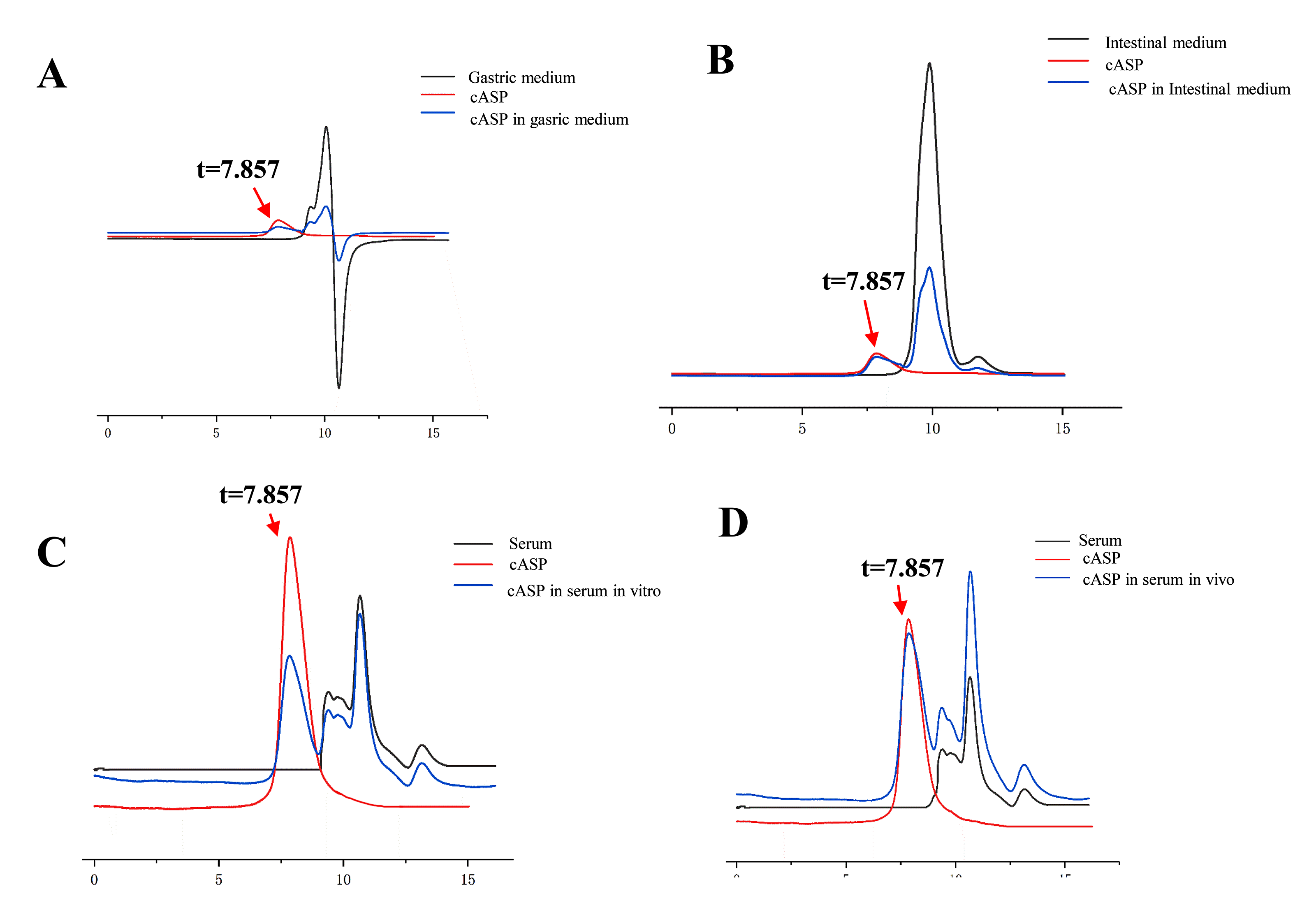


**Fig S1.** HPSEC chromatogram of the cASP. A) HPSEC chromatogram of the cASP after *in vitro* gastric digestion for 24 h. B) HPSEC chromatogram of the cASP after *in vitro* intestinal medium digestion for 24 h. C) HPSEC chromatogram of the cASP after *in vitro* serum digestion for 24 h. D) HPSEC chromatogram of the cASP after *in vivo* serum digestion for 2 h.
